# Atypical modulation of electrodermal reactivity during exposure to graded unisensory and multisensory stimuli in autistic children and adolescents

**DOI:** 10.3389/fpsyt.2026.1783075

**Published:** 2026-03-19

**Authors:** Sandra Brouche, Natalie Rigal, Christian Paroissin, Nora Bouaziz, Jean-Marc Baleyte, Faby Cazalis-Judet de la Combe

**Affiliations:** 1 Université Paris Cité, Institut de Psychologie, Laboratoire de Psychopathologie et Processus de Santé, Paris, France; 2 Université Paris Nanterre, Département de psychologie, UR Clipsyd, Nanterre, France; 3 Unviersité de Pau et des Pays de l’Adour, CNRS, Laboratoire de Mathématiques et de leurs Applications de Pau (LMAP), Pau, France; 4 Maison de l’Enfant et de la Famille, Centre Hospitalier Intercommunal de Créteil, Créteil, France; 5 École des Hautes Études en Sciences Sociales, Centre National de la Recherche Scientifique (CNRS), Centre d’Analyse et de Mathématique sociales, Paris, France

**Keywords:** autism, children/adolescents, electrodermal reactivity, multisensory, sensory processing, unisensory

## Abstract

**Background:**

Despite the substantial increase in research on sensory processing in autism, the standardization of their assessment remains non-consensual. Physiological measures such as electrodermal reactivity (EDR) are still rarely used in clinical practice, although previous studies have reported differences in reactivity between autistic individuals and the general population. This experimental study aimed to test electrodermal reactivity (EDR) as an objective and measurable marker of sensory perception in children and adolescents with autism.

**Methods:**

Data were collected from 37 participants, including 21 typically developing children (11 girls and 10 boys, M = 9.86 years) and 16 autistic children (all boys, M = 11.37 years). EDR was assessed both in its tonic (SCL) and phasic (SCR) components. Participants were exposed to three types of stimuli: unisensory (visual or auditory) and multisensory (audiovisual). Each stimulus was presented at increasing intensity from the first to the third exposure (nine conditions in total).

**Results:**

SCL was consistently lower in autistic participants as compared to typically developing peers. SCR was lower in autistic participants for 4 exposure conditions out of 9. In contrast, no between group differences were found in relation to stimulus intensity or between unisensory and multisensory modalities. Within-group comparisons revealed significant increases in SCR during stimulus conditions only in the control group. Additionally, no significant age-related effects were observed.

**Conclusions:**

These findings highlight a specific EDR pattern in autistic children and adolescents during sensory stimulus processing characterized by hypoactivity in both tonic and phasic components of the signal. Future research should aim to better characterize this profile using a broader range of stimuli, across varied exposure contexts, and by integrating these physiological measures with cognitive assessments.

## 1 Introduction

Sensory differences are included among the diagnostic criteria for autism in the DSM-5-TR (criterion B4) (1). This diagnostic evolution is the result of 80 years of research, initiated by Kanner’s first description of autism, which highlighted the specificities of sensory perception in this population (2, 3). It also reflects the accounts of families and autistic individuals, who have emphasized the significant impact of these differences on daily life (4–6). Sensory perception differences represent a major issue as they influence the development, quality of life, and autonomy of autistic individuals (7–9). This recognition is reflected in the number of studies published per year, which has increased by an average of 16% per year between 2010 and 2025^1^. Notably, significant progress has been made regarding the assessment of sensory perception in autism, particularly through systematic reviews and experimental studies that have shed light on each tool and technique used (10–12). The absence of clear diagnostic markers to characterize sensory processing differences could lead to considerable variability in evaluations. To address these discrepancies, He et al. (13) recently proposed a five-level classification of sensory perception, which includes: 1. sensory-related neural excitability, 2. perceptual sensitivity, 3. physiological reactivity to sensory input, 4. affective reactivity to sensory input, and 5. behavioral responsivity to sensory input. This taxonomy offers a structured framework to analyze the complexity of sensory processes and helps standardize terminology in the field, which is particularly valuable given the current lack of consensus (14, 15). As the authors explain, the same term can often encompass vastly different realities.

Physiological reactivity goes beyond behavioral observations by providing objective measurements of an individual’s direct response to one or more stimuli. Specifically, heart rate, electrodermal reactivity (EDR), eye gaze and muscle response are four biomarkers that have shown significant differences in autistic individuals and those with typical development (16–19). However, studies utilizing these so-called “functional biomarkers” rely varied methodologies and stimuli, making it challenging to synthesize findings on the subject (20). EDR, as a particularly sensitive measure of changes in sympathetic nervous system (SNS) activation in response to sensory stimuli, was selected as an indicator in this study (21).

EDR is characterized by two main components: the tonic response (known as SCL, or skin conductance level), and the phasic component (known as SCR, or skin conductance response) (22, 23). SCL reflects the baseline level of skin conductance, whereas SCR captures transient changes in conductance triggered by specific stimuli. Additional markers, such as SCR amplitude and peak values, are often used to further characterize EDR. Amplitude is defined as the difference between the peak conductance level and the baseline level immediately preceding the response (22), providing an index of the intensity of the autonomic nervous system’s reaction to a stimulus. Peak values refer to the highest level of electrodermal activity reached following stimulus presentation.

Importantly, electrodermal responses are not static over time. Repeated exposure to a stimulus may lead to different patterns of physiological response, including habituation or sensitization (22). Although habituation and sensitization have been well documented in typically developing populations, particularly using electrodermal measures, these processes remain understudied in autistic children, to our knowledge. In addition to repetition effects, electrodermal activity is sensitive to variations in stimulus intensity and cognitive load. Progressive increases in stimulation or task demands have been associated with elevated EDR in typically developing individuals (24).

Beyond these within-individual response patterns, studies comparing autistic and typically developing individuals have reported group differences in overall EDR levels. A meta-analysis on the subject has highlighted conflicting findings, with some studies showing heightened EDR in autistic individuals, while others report diminished responses to sensory stimuli (20). This discrepancy in findings can be partly attributed to variations in study contexts, particularly the nature of the stimuli used. For example, studies can involve either a single sensory channel, such as auditory tones, visual flashes, or tactile stimulation (25–28), or multiple sensory channels, such as play-based paradigms, social interaction contexts or virtual reality environment (16, 29, 30). Multisensory approaches are designed to replicate sensory experiences in daily life, but they also introduce additional variables that can complicate the interpretation of EDR responses.

Nevertheless, a substantial amount of research suggests differences in multisensory integration at both behavioral and neural level (31–34). Then, investigating multisensory stimulus processing through EDR is necessary, as it could reveal differences in autistic people. Notably, the “multisensory prior entry effect” refers to the observation that multisensory stimuli are processed more quickly than unisensory stimuli (35, 36). This phenomenon is attributed to increased selective attention directed toward multisensory stimuli, which facilitates their processing by the brain (37, 38). Cognitive studies measuring reaction times have shown that this effect is reduced in autistic individuals (36, 38–42). However, to date, there are no physiological data on this effect in autistic individuals.

From a developmental perspective, studies have shown higher EDR in children compared to adults (43, 44). This heightened EDR sensitivity in younger individuals may be attributed to differences in the maturation of their autonomic nervous system (ANS) (45). In young children, the ANS is still developing, leading to more pronounced physiological reactivity to sensory stimuli. However, although this reactivity tends to decrease with age, EDR can still be influenced by various emotional and situational factors in adults. To date, to our knowledge, no study has yet explored age-related differences in EDR responses among autistic individuals.

The objective of this experimental study was to explore differences in physiological reactivity to sensory stimuli between autistic children and adolescents and typically developing peers. Physiological reactivity was assessed using a quantitative measure: electrodermal reactivity (EDR). The following hypotheses were tested:

1. Autistic children are expected to exhibit enhanced EDR compared to typically developing children, whether in response to unisensory or multisensory stimuli. This hypothesis is based on previous studies that have shown physiological differences between these two groups, which may be associated with heightened ANS reactivity in autistic children (20, 29).
2. The progressive increase in the intensity of sensory stimuli across successive exposures leads to a corresponding increase in EDR in both groups of children. Previous studies have shown that continuous processing of stimuli, particularly in situations involving mental effort, is associated with an increase in EDR in typically developing individuals (24). It remains to be determined whether this phenomenon occurs in a similar way in autistic children.
3. In typically developing children, EDR is at a lower level in response to multisensory stimuli compared to unisensory stimuli, reflecting better integration of multisensory information. In contrast, it is likely that this difference is not observed in autistic children, as cognitive studies have shown distinct processing of multisensory stimuli in this population (36, 38–42).
4. EDR decreases with age in typically developing children, in relation to the maturation of the autonomic nervous system (ANS). This decrease may not be observed in autistic children, as their nervous system maturation may follow a different trajectory (43, 44).

## 2 Method and materials

### 2.1 Participants

Forty-two children and adolescents participated in this study (*n* = 21 autistics, *n* = 21 controls; see **Table 1**). Following electrodermal signal analysis, data from five participants were excluded due to unusable results (signal loss caused by movement or signal below reliability thresholds). Descriptive comparisons indicated that the excluded participants did not differ from the rest of the sample in terms of developmental profile, cognitive functioning, or type of care received. As a result, the final sample comprised 37 participants, including 16 children with autism (*n* = 16 boys; *M*_age_ = 11.37, *SD* = 3.4; range = 7-17) and 21 typically developing children (*n* = 11 girls and *n* = 10 boys; *M*_age_ = 9.86, *SD* = 2.6; range = 6-14). The inclusion criteria for study participants were as follows: age between 6 and 17 years, no genetic disorders (such as fragile X syndrome, Down syndrome, or Williams syndrome), and no history of epilepsy. The minimum age of 6 years was chosen to ensure participants’ ability to understand instructions and staying engaged. Autistic children and adolescents were required to have a diagnosis confirmed by a psychiatrist or pediatric psychiatrist prior to participation. Nine participants presented one or more neurodevelopmental comorbidities, including attention-deficit/hyperactivity disorder (ADHD), social communication disorder, learning disorders, motor disorders, and speech disorders (n = 7 autistic participants, n = 2 controls). Four participants had one or more psychiatric comorbidities, including social anxiety, specific phobia, obsessive-compulsive disorder (OCD), and panic disorder (n = 2 autistic participants, n = 2 controls) (see **Table 1** for details). All participants had normal visual and auditory acuity, as characterized by parents’ and/or participants’ reports.

**Table 1:** Descriptive statistics of participant characteristics by group.

N = 37 Sex (Boy : Girl)	Autistic (n = 16) 16:0			Control (n = 21) 10:11		
Groups	M	SD	Range	M	SD	Range
Age (years)	11.4	3.4	(7.0 – 17.0)	9.7	2.6	(6.0 – 14.0)
Verbal IQ^a^	88.7	21.0	(59 – 121)	111.9	10.6	(86 – 127)
Neurodevelopmental comorbidity^b^	7			2		
Psychiatric comorbidity^c^	2			2		
First words age (months)	26.0	13.9	(6 – 60)	13.7	3.9	(9 – 22)
First steps age (months)	18.3	10.9	(8 – 48)	13.2	1.9	(9.5 – 17)
Healthcare^d^	16			4		
Medical treatment	3			2		
Sleep difficulty	6			2		
Food selectivity	7			3		

^a^ Subtests Similarities and Vocabulary (WISC-V). ^b^ Neurodevelopmental comorbidity: attention-deficit/hyperactivity disorder (ADHD; 4 autistics, 2 controls), social communication disorder (2 autistic), learning disorders (4 autistics), motor disorders (3 autistics), speech disorders (2 autistics). ^c^ Psychiatric comorbidity: social anxiety (2 autistics, 1 control), specific phobia (1 control), obsessive-compulsive disorder (OCD; 1 autistic, 1 control), panic disorder (1 autistic). ^d^ Care: number of children receiving care from a healthcare professional in the medico-social sector.

### 2.2 Recruitment

Autistic children and adolescents were recruited through three institutions: The *Centre Hospitalier Intercommunal de Créteil*, the *Fondation Vallée* and the TEDIS study group (46). Participants with typical development were recruited from the LabSchool Paris (https://www.labschool.fr/) (47, 48).The study received ethical approval from the *Comité de Protection des Personnes* (CPP; French Ethics Committee for the Protection of Individuals; N° 2022-022000-97) and was conducted in accordance with the principles of the Declaration of Helsinki. Written informed consent/assent was obtained from all participants or their legal guardians. An information sheet, tailored to the child’s level of understanding, was developed in the form of a comic strip (https://osf.io/a7g84/files/osfstorage).

### 2.3 Protocol and material

#### 2.3.1 Experimental application

An application was developed exclusively for this project. It was designed to automate the collection of sociodemographic information and to present the sensory stimuli. Results were automatically exported in Excel format to the “Documents” folder on the computer. InTEnSeA^©^ was developed in C++ using QT framework version 6 (49). The application is licensed under the CeCILL license. Executable files for Mac and Windows are available on GitHub (https://github.com/sbrouche/InTEnSeA) and the experimental stimuli are hosted on OSF (see Data Availability section).

#### 2.3.2 Sensory stimuli

Visual, auditory, and audiovisual stimuli were displayed on a *ViewSonic* TD2230 22” touchscreen monitor (CE-certified) with a resolution of 1920 x 1080 pixels. The screen was positioned 60 cm from the participants, at eye level. The stimuli were developed based on sensory codes identified during a qualitative study on sensory processing descriptions from parents of autistic individuals (8). These stimuli, specifically designed for this protocol, were presented to participants with a progressive increase in stimulation across three exposures, following a parametric design. The classification of exposure levels (low = 1, moderate = 2, high = 3) was guided by the incremental addition of sensory elements known to increase cognitive and sensory load (*e.g.*, spatial complexity, social cues, or sudden changes), as identified in the qualitative data. The level of exposure corresponds to the complexity and accumulation of stimuli rather than to changes in physical parameters (*e.g*., luminance or sound amplitude), which were kept constant across conditions. For visual stimuli, participants viewed a moving landscape as seen through the window of a moving train. Three one-minute videos were presented: (V_1_) the first depicted a moving landscape; (V_2_) the second added alternating visual fields (near/far) to the moving landscape; (V_3_) the third introduced light variations combined with the alternating visual fields. For the auditory stimuli, participants wore headphones and were exposed to ambient sounds recorded inside a train, including conversational speech and background noise. Three one-minute audio recordings were presented: (A_1_) the first played the background noise of a moving train; (A_2_) the second included a conversation between passengers; (A_3_) the third combined the previous two with the addition of a disruptive phone ringtone. Finally, for the audiovisual stimuli, a series of three one-minute videos combining the visual and corresponding auditory recordings was presented (AV_1_, AV_2_, AV_3_).

#### 2.3.3 Electrodermal reactivity

The eSense Skin Response Mindfield^®^ (battery-powered, CE-certified) was used in this study to measure electrodermal signals during the presentation of sensory stimuli. Electrodermal activity was recorded using disposable palmar gel electrodes from Mindfield^®^ and 3M Red Dot™, specifically designed for children. These electrodes were applied to the non-dominant hand of participants, positioned on the hypothenar and thenar regions. The measured index represented the Sympathetic Skin Response (SSR), expressed in microsiemens (μS). The device was connected to a smartphone or tablet for data acquisition, with a sampling frequency of 5 Hz. All data were subsequently exported in CSV format. A one-minute baseline was recorded for each participant at the beginning of the test, followed by exposure to sensory stimuli, each lasting one minute. Electrodermal responses were recorded during each exposure, resulting in a total of nine measurements per participant.

#### 2.3.4 Procedure

**Figure 1:**
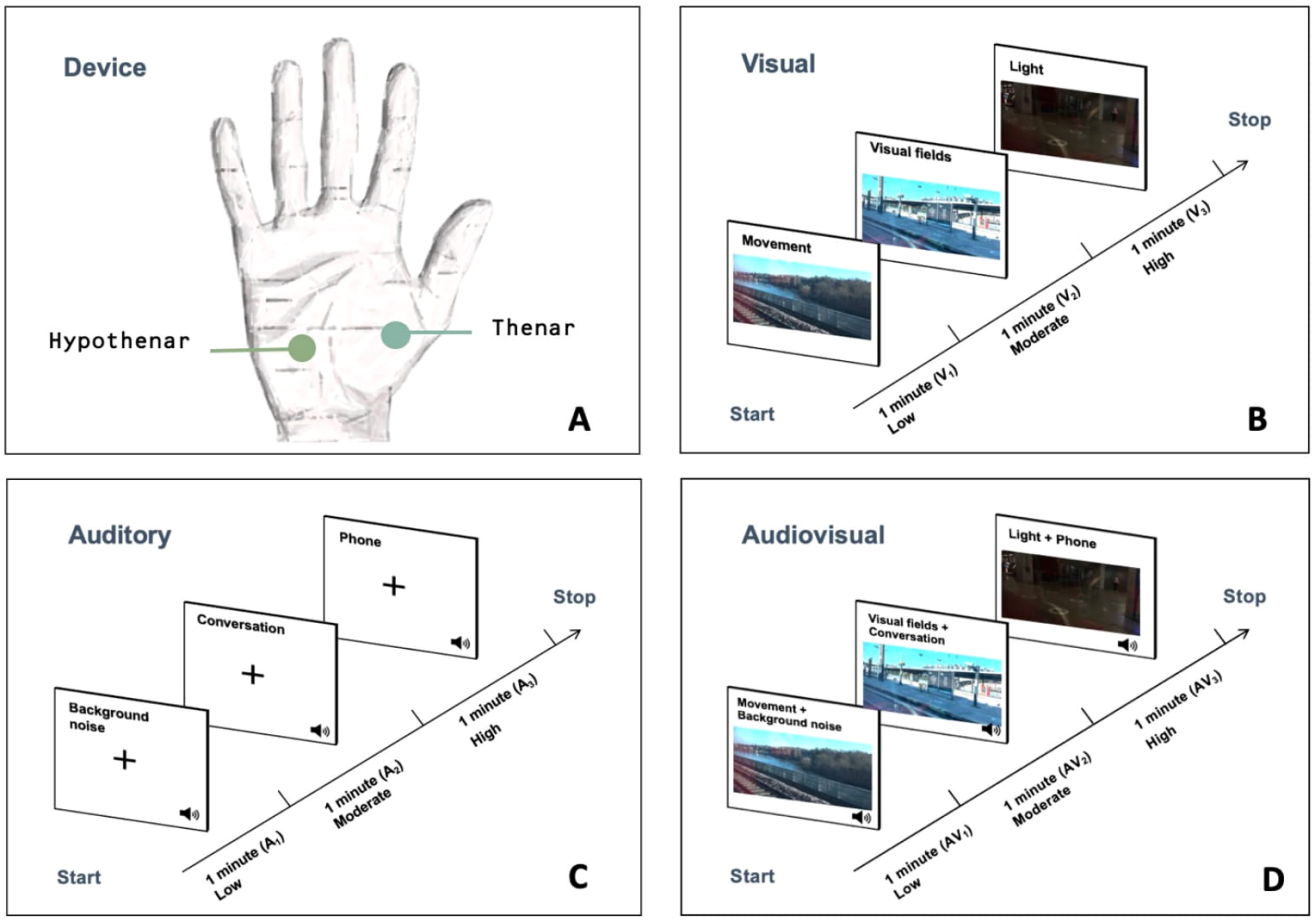
Procedure for administering sensory stimuli. **(A)** Illustration of the participant’s setup facing the screen. **(B)** V_1_ = movement; V_2_ = addition of a near/far visual field alternation; V_3_ = addition of light variation. **(C)** A_1_ = background sound; A_2_ = Addition of discussion of nearby individuals; A_3_ = addition of an unexpected smartphone ringtone. **(D)** = AV_1_ = V_1_ + A_1_; AV_2_ = V_2_ + A_2_; AV_3_ = V_3_ + A_3_.

Sociodemographic questionnaires were completed by the participants’ parents. The entire testing session lasted approximately one hour for the children. A familiarization period with the equipment, lasting 10 to 15 minutes, was provided. During this phase, children were introduced to the equipment (electrodes and monitor), allowed to touch the materials, and received a step-by-step explanation of the procedure in simple, age-appropriate language. For autistic children, an additional meeting could be arranged to reduce stress associated with the novel testing environment. The testing environment was kept quiet and minimally stimulating, with limited visual and auditory distractions. Parents were allowed to remain present in the room if this facilitated the child’s comfort. The session began with the administration of the verbal IQ test using the Wechsler Intelligence Scale for Children - Fifth Edition, focusing on the Verbal Comprehension Index (approximately 15 minutes), followed by the placement of electrodes and the presentation of sensory stimuli (30 minutes) (see **Figure 1a**). For younger children, a model of the electrodes was demonstrated on a teddy bear or a plush panda before placing them on the child’s hand. Children could stop the test at any time using a predefined gesture. If the investigator observed significant signs of agitation, stress, or discomfort, the task was immediately stopped. Overall, children responded well to the procedure. Most showed curiosity and were amused by the equipment. However, two children were unable to remain still during the session, which led to its interruption. First, a baseline recording was conducted. If no anomalies were detected in the signal and the child was comfortable with the equipment, the test continued (see **Figures 1b-d**). Each exposure lasted one minute and was presented consecutively without breaks. The investigator monitored compliance throughout the task and provided standardized reminders only if necessary. At the end of the test, participants were thanked for their involvement and received a small gift bag containing a pen, a notebook, a sensory ball, and a participation pin. The standardized procedure, including the verbal instructions provided to children, is described in the **Supplementary Material 1**.

#### 2.3.5 Electrodermal signal processing

The electrodermal signal was processed using *Ledalab* V3.4.9, a *Matlab*-based software for skin conductance data analysis (50, 51). For each participant, two files were imported and processed in *Ledalab*: one file containing the baseline data and another with the signal during exposure to sensory stimuli. The data were preprocessed by applying a smoothing technique (window width 8, Gaussian window type) and manually correcting artifacts using the graphical interface. A Continuous Decomposition Analysis (CDA) was applied to decompose the tonic and phasic phases of the signal (50). Four parameters were extracted from the signal for analysis (see **Table 2**). The results were then exported in CSV format.

**Table 2:** Definitions of the four electrodermal parameters.

Electrodermal parameters	Definitions
Skin Conductance Level (SCL)	Represents the baseline level of sympathetic nervous system activity
Skin Conductance Response (SCR)	Reflects the activation of the sympathetic nervous system in response to specific stimulation
SCR Amplitude	Difference between the conductance value immediately before the stimulus and the peak value recorded afterward
Peak rates (per minute)	Number of distinct fluctuations in skin conductance per minute

Definition based on Boucsein (22).

### 2.4 Data analyses

An RData file containing the values of the four electrodermal parameters for each participant was imported into *RStudio* (version 2024.04.2 + 764) using *R* (version 4.4.1) for statistical analyses. We used an independent groups design with repeated measures (nine exposures and one baseline). A between group analysis (autistic vs control) and a within-group analysis (differences between exposures) were conducted for the four dependent variables/parameters.

To determine the most appropriate statistical test to compare participants characteristics (age, verbal IQ, age of first steps, and age of first words), the normality of the distributions was checked using both empirical and graphical methods. Given the assumption of non-normality in the data and the small sample size, we chose a non-parametric test: the Wilcoxon-Mann-Whitney test to examine between-group differences.

**Figure 2:**
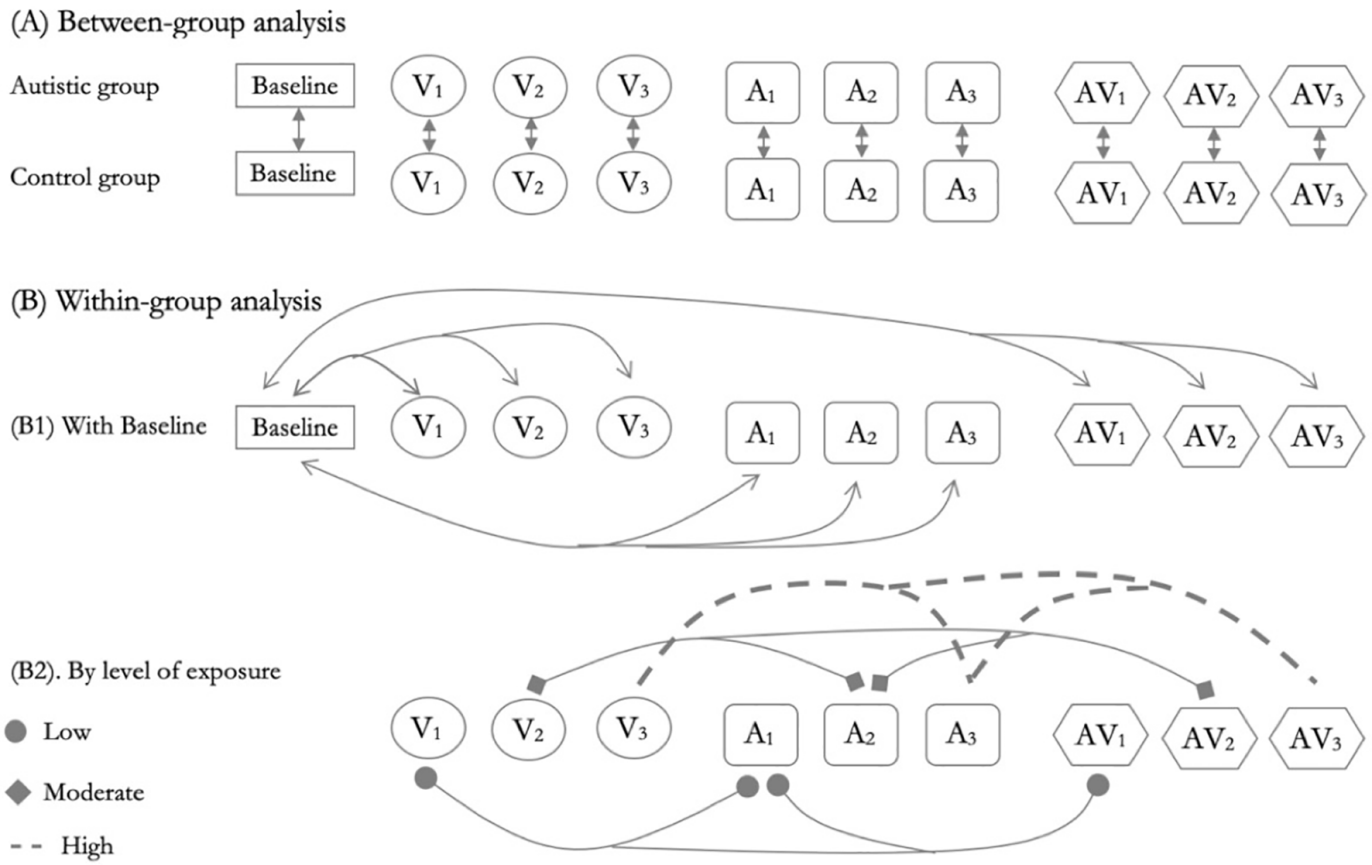
Overview of statistical comparisons across sensory conditions and exposure levels for autistic and control groups. **(A)** This section illustrates the between-group analyses (autistic vs. control participants) for each stimulus condition. **(B)** This section shows the within-group comparisons, including the baseline and comparisons according to the levels of sensory exposure. V= visual, A = auditory, AV = audiovisual, 1 = low, 2 = moderate, 3 = high.

Electrodermal signals (SCL and SCR) were analyzed using Functional Data Analysis (FDA). Between-group and within-group differences were assessed using the FP-test implemented in the fdANOVA package (52). This permutation test, based on a basis function representation of the data, does not assume normality and is particularly suited to detecting global differences in functional data, especially in studies with small sample sizes. **Figure 2** illustrates the comparisons made across stimulus exposures, both between and within groups.

The impact of baseline correction on the results was examined. As it did not alter the response patterns, raw signals were used to preserve dynamic precision.

Since peaks and SCR amplitude are not continuous data, the non-parametric Wilcoxon-Mann-Whitney test was used to assess between-group differences, and the Friedman test to assess within-group differences.

To further explore potential age-related effects in SCL and SCR signals, an exploratory analysis was conducted by testing all possible splits of participants into two age groups, provided that each subgroup contained at least two participants. Each split was tested independently. To control for inflation of type I error due to multiple testing, Holm correction was applied across all tested splits.

Finally, to examine the association between Verbal IQ scores and EDR, Spearman correlation analyses were conducted for the four electrodermal parameters. False discovery rate (FDR) correction was applied to control for multiple comparisons.

## 3 Results

### 3.1 Participants characteristics

No significant age difference was observed between the two groups, although children with autism were on average 19 months older than typically developing children (*U* = 217, *p* = .130, *r* = .25). Differences between groups were observed for “age of first steps” and “verbal IQ” with lower scores for autistic children and adolescents (age of first steps: *U* = 102, *p* = .038, r = .34; verbal IQ: *U* = 53.50, *p* <.001, *r* = .52). The difference in first steps corresponded to an average delay of 5.1 months in the autistic group. Finally, no difference was found for the variable “age of first words” (*U* = 224.50, *p* = .081, *r* = .29).

### 3.2 Between group differences in EDR during baseline

The results of the permutation FP-test indicated a significant group difference in SCL, with lower signal levels recorded in autistic children and adolescents compared to those with typical development (*p* = .017), and a similar pattern was found for SCR (*p* = .043). The Wilcoxon-Mann-Whitney test confirmed this trend for SCR amplitude (*U* = 85, *p* = .011, *r* = .42). In contrast, no significant difference in average peak rates during the baseline between the two groups was found between the two groups (*U* = 228, *p* = .068, *r* = .30).

### 3.3 Between group differences in EDR during test

Regarding the unisensory conditions (visual and auditory), all SCL signals showed a significant difference between the two groups with lower signals found in children and adolescents in the autistic group compared to those in the control group (see **Table 3**). For the SCR signal, a significant difference was observed in V2 and A1. SCR response amplitudes did not differ significantly, except for two exposures (V1 and A3). Multisensory conditions yielded similar patterns: significant group differences in SCL signals, and significant differences in SCR signal and SCR response amplitudes for the first two conditions (AV1 and AV2). No differences between the two groups were observed in peak rates across all conditions (see **Table 3**).

**Table 3:** Results of the functional permutation test (FP-test) and Wilcoxon-Mann-Whitney U-test comparing the autistic and control groups on the four electrodermal parameters.

Sensory stimuli exposition				Electrodermal parameters			
				p-value (autistic vs. control)			
		FP test		Wilcoxon-Mann-Whitney			
		SCL	SCR	Amplitude (W)	r	Peak rate (W)	r
Visual	V1	.006**	.182	.021* (92)	.38	.154 (215)	.23
	V2	.001***	.012*	.382 (139)	.14	.297 (133.5)	.17
	V3	.003**	.231	.156 (121)	.23	.581 (149.5)	.09
Auditory	A1	.005**	.007**	.073 (109)	.30	.570 (187)	.09
	A2	.013*	.133	.059 (106)	.31	.866 (162)	.03
	A3	.005**	.153	.019* (91)	.39	.783 (158.5)	.05
Audiovisual	AV1	.004**	.007**	.022* (93)	.38	.319 (135)	.16
	AV2	.001***	.014*	.028* (96)	.36	.613 (151)	.08
	AV3	.001***	.232	.101 (114)	.27	.250 (130)	.19

Significance levels: p <.05*, p <.01**, p <.001***.

### 3.4 Within-group differences in EDR (baseline and test)

#### 3.4.1 Autistic group

**Figure 3:**
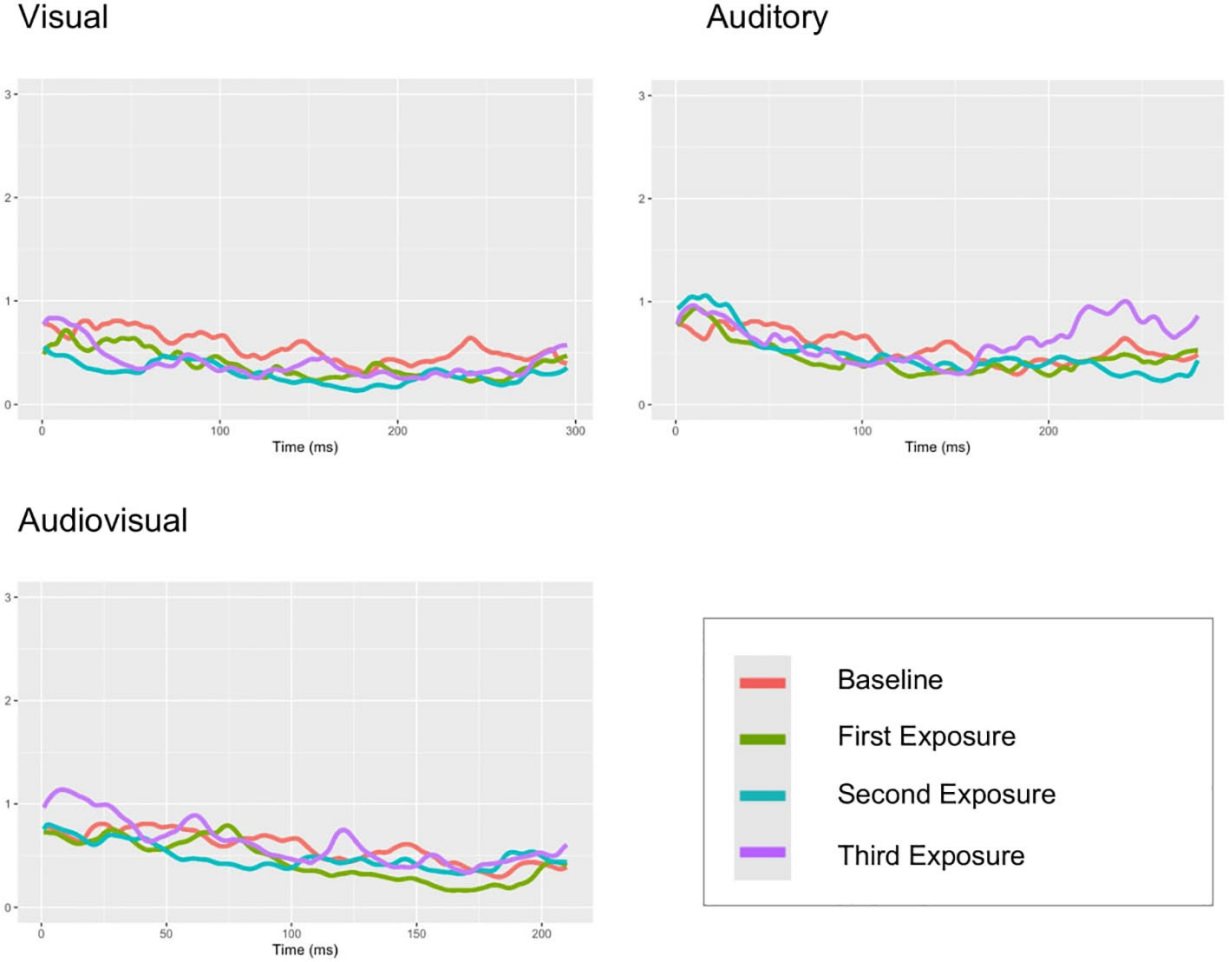
Mean SCR signal plots for each stimulus type (visual, auditory, and audiovisual) and the baseline in children and adolescents from the autistic group.

Regarding SCR, the permutation FP-test showed a significant difference between the baseline and the visual exposure at moderate intensity (see **Table 4**). No significant differences were found between the baseline and the auditory or audiovisual exposures (see **Figure 3**).

**Table 4:** Results of the functional permutation test (FP-test) for within-group comparisons in autistic and control groups.

FP-test (*p*-value)							
SCR	Autistic	Control		SCL	Autistic	Control	
Baseline							
Baseline-V1	.261		.003**		.879		.352
Baseline-V2	.022*		.000***		.559		.436
Baseline-V3	.252		.000***		.607		.503
Baseline-A1	.610		.000***		.978		.176
Baseline-A2	.571		.012*		.884		.265
Baseline-A3	.431		.002**		.694		.137
Baseline-AV1	.433		.000***		.597		.059
Baseline-AV2	.591		.000***		.805		.066
Baseline-AV3	.700		.000***		.512		.013*
Low							
V1-A1	.760		.021*		.880		.621
V1-AV1	.719		.071		.535		.239
A1-AV1	.578		.694		.646		.295
Moderate							
V2-A2	.082		.092		.508		.760
V2-AV2	.033*		.116		.444		.294
A2-AV2	.782		.763		.982		.462
High							
V3-A3	.100		.031*		.419		.342
V3-AV3	.109		.113		.286		.067
A3-AV3	.738		.512		.814		.336

Comparisons between levels of sensory load revealed no difference between low-load conditions, a significant difference between visual and audiovisual stimuli under moderate load, and no difference under high load.

For SCL, no significant differences were found between the baseline and any of the three sensory modalities, nor across the different levels of sensory load.

The Friedman test indicated a significant main effect of condition for SCR amplitude (*χ*² (9) = 29.85, *p* <.001). However, *post hoc* Wilcoxon tests with Bonferroni correction revealed no significant pairwise differences between the load levels within modalities. Finally, no significant differences were found across conditions for peak rate (*χ*² (9) = 14.55, *p* = .104).

#### 3.4.2 Control group

**Figure 4:**
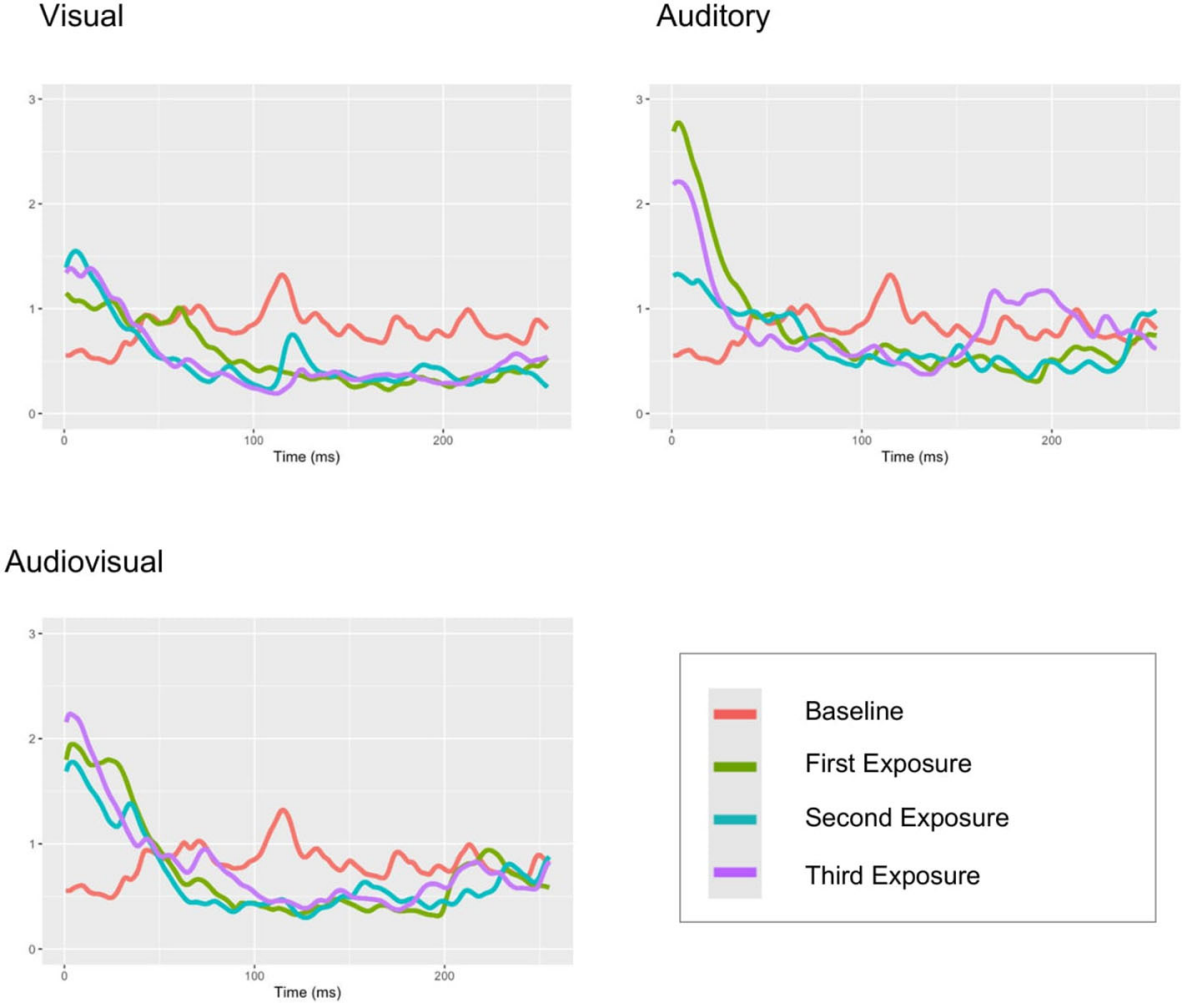
Mean SCR signal plots for each stimulus type (visual, auditory, and audiovisual) and the baseline in children and adolescents from the control group.

For the control group, significant differences in SCR were observed between the baseline and all stimulus condition: visual, auditory and audiovisual (see **Figure 4** and **Table 4**). Comparisons across stimuli types showed significant differences in SCR between the visual and auditory conditions under low load (V1/A1: *p* = .021) and high load (V3/A3: *p* = .031), but not under moderate load.

For SCL, a significant difference was found only between the baseline and the audiovisual condition under high load (Baseline/AV3: *p* = .013).

For SCR amplitude, the Friedman test indicated significant differences across the nine experimental conditions (*χ*² (9) = 35.51, *p* <.001). *Post hoc* analyses showed significant differences from baseline to the visual condition (Baseline/V2: *p* = .015; Baseline/V3: *p* = .013), but no significant differences between stimulus types.

A significant effect was found for peak rate (*χ*² (9) = 18.16, *p* = .033), but *post-hoc* comparisons did not show significant differences between levels of sensory load.

### 3.5 Age differences in EDR

In the autistic group, nominally significant age-related differences were observed for two stimuli in SCL and two in SCR prior to correction for multiple comparisons. For SCL, exposures to V2 and AV2 showed differences between participants aged 7–9 and 10–17 years (V2: *p* = .032; AV2: *p* = .020). For SCR, a difference at baseline was observed when comparing children aged 7–8 with those aged 9–17 years (*p* = .003). Finally, for exposure to A1, a difference was found between participants aged 7–9 and 10–17 years (*p* = .024). However, after applying Holm correction across all tested age splits, none of these differences remained statistically significant (see **Supplementary Material 2**). In the control group, no significant age-related differences were found for any stimulus or signal (SCL or SCR).

### 3.6 Correlation between Verbal IQ and EDR

Correlation analyses were conducted to examine the association between Verbal IQ scores and the four electrodermal reactivity parameters. With the exception of a single isolated finding (Amplitude V1), no significant correlations were observed, regardless of group or condition (all adjusted p >.05; see **Supplementary Material 2**). These findings indicate that EDR was not significantly associated with verbal cognitive functioning in the present sample.

## 4 Discussion

This study aimed to explore differences in physiological reactivity between autistic children and typically developing peers exposed to sensory stimuli varying in modality and intensity. Participants completed a task involving the processing of unisensory and multisensory stimuli at varying intensities. Results revealed significant group differences in EDR. Specifically, autistic children exhibited significantly lower tonic responses (SCL) across all stimulus conditions. In contrast, phasic responses (SCR) were significantly lower in the autistic group in only a subset of conditions. No significant effects of stimulus modality (unisensory, multisensory) or intensity were observed within either group. However, a dissociation was observed between groups in the evolution of the phasic response from baseline to stimulus exposure: typically developing children showed significant increases in SCR, indicating a clear physiological reactivity to sensory input. In contrast, no such difference was observed in the autistic group, indicating a blunted or less differentiated phasic response. Finally, analysis of age-related effects in EDR revealed no notable effects in either group.

The first key finding of the study concerns electrodermal responses during the processing of sensory stimuli at baseline. Mean electrodermal activity was significantly lower in autistic children compared to their typically developing peers across all electrodermal parameters, except for peak rates. This finding is consistent with a body of literature reporting reduced electrodermal activity at rest in autistic people (20). However, there are discrepancies across studies regarding the specific characteristics of this response. For example, the study by Eilam-Stock et al. (17) highlighted this difference in autistic adults, but only at the level of phasic SCR responses. In contrast, the study by Schoen et al. (53) identified this difference in autistic children, specifically in tonic SCL responses. Our results suggest that autistic children and adolescents exhibit hypoactivity of the autonomic nervous system (ANS) at rest, supporting the hypothesis of altered autonomic regulation across three electrodermal parameters: tonic SCL responses, phasic SCR responses, and SCR amplitude. A reduced electrodermal response has also been reported in other clinical populations. A systematic review highlighted lower EDR at rest in individuals with ADHD (54), particularly in females, consistent with hypoarousal models (55). Findings in schizophrenia appear more heterogeneous, with reports of both hypo- and hyper-responsive electrodermal response (22). These disorders, although characterized by distinct etiologies, have been described in the literature as involving alterations in autonomic regulation, raising the question of whether disorder-specific electrodermal signatures may exist. Comparative studies would be necessary to determine whether EDR reflects shared regulatory mechanisms or physiological patterns specific to each disorder.

Regarding within-group analyses, exposure to different sensory processing conditions confirmed the consistency of the reactivity difference between the two groups of children. Typically developing children showed significant differences in phasic SCR responses between baseline and stimulus exposure. These responses were characterized by an initial increase in SCR during the first seconds of exposure, followed by a gradual decrease in signal intensity, which may suggest a habituation process as described by Boucsein (56). This difference was not observed in autistic children and adolescents, which may be related to the overall lower EDR observed in this group. These findings are in line with a body of research reporting reduced EDR in autistic individuals compared to their typically developing peers (17, 26, 53, 57). However, these findings remain subject to debate: some studies have reported heightened EDR (27, 29), while others have found no difference (30). The literature also presents mixed results regarding whether EDR is higher or lower in autistic children compared to autistic adults.

In light of these findings, our data support the hypothesis of a reduced response to sensory stimuli, suggesting a difference in sympathetic nervous system (SNS) regulation in autistic individuals. The SNS plays a central role in modulating reactivity to sensory stimuli by adjusting sensitivity to environmental changes. This specificity could explain why autistic individuals tend to exhibit unusual reactions to sensory stimuli and may be more prone to sensory overload (58–60). These manifestations may be related to less efficient integration of sensory input, which could limit the ability to modulate exposure thresholds. Sensory Integration Theory describes such anomalies in the modulation of sensory information (61). Ayres proposed that dysfunctions within regulatory systems underlie difficulties in organizing and integrating input from multiple sensory channels. However, in her original formulation, she did not explicitly address the role of the ANS (62). Subsequent research later examined this dimension and demonstrated that sensory modulation involves autonomic mechanisms (63).

The processing of unisensory and multisensory stimuli did not reveal any significant differences in electrodermal reactivity in either group. This finding contrasts with the literature on the multisensory prior entry (MPE) effect (37, 38). The MPE effect, traditionally measured through reaction time, suggests that multisensory processing enhances response speed. Several studies have shown that this effect is less pronounced in autistic people (36, 39–42). However, the absence of group differences observed in EDR may be explained by the fact that this physiological measure primarily reflects ANS activity (21–23, 64), which is generally influenced by emotional salience and arousal. In contrast, the processes involved in the MPE effect are primarily attentional. Changes in cognitive load associated with shifts in sensory modality may not be strong enough to result in a detectable variation in electrodermal responses. A more in-depth analysis of this phenomenon may require combining cognitive and physiological measures. A follow-up study is currently underway using the same stimuli to evaluate their effect on working memory and selective attention in typically developing children and adolescents.

Finally, the results regarding age-related changes in EDR did not support the proposed hypothesis. Contrary to expectations, no significant age-related decrease in EDR was observed in either typically developing or autistic children. This finding runs counter to previous literature showing higher EDR levels in children compared to adults (43, 44). It could be partly explained by the use of stimuli that were not suitable for detecting this type of difference, or by a genuine absence of age-related effects on ANS reactivity. Future research should include comparisons with adults and vary the nature of the stimuli to better capture the impact of development on EDR.

Several limitations must be considered in order to adequately interpret these results. First, our sample exhibited a significant gender imbalance, with an overrepresentation of males in the autistic group, while the control group included participants of both sexes. This difference may be explained by the well-known bias towards males in autism diagnosis, leading to their overrepresentation in institutional settings (65). Given that electrodermal reactivity may vary as a function of biological sex (22) and considering the documented differences in autistic phenotype between males and females (66), we cannot assume that the present findings are generalizable to individuals presenting a female autism phenotype. Investigating electrodermal reactivity in autistic females therefore represents an important direction for future research, both to better characterize their clinical profile and to determine whether sensory reactivity differs from that observed in autistic males. Furthermore, the small sample size limits the generalizability of our conclusions. Second, inclusion in the autistic group was based on a clinical diagnosis confirmed by a specialist. However, no information was collected regarding gold-standard diagnostic measures (e.g., ADI-R, ADOS-2) or DSM-5-TR severity levels (Levels 1, 2, or 3), limiting the characterization of clinical heterogeneity within the group. In the absence of standardized severity measures, we conducted additional correlation analyses between Verbal IQ scores and electrodermal reactivity to explore whether cognitive level might account for autonomic variability. These analyses did not reveal any significant associations, suggesting that the observed autonomic differences were not explained by verbal level in our sample. Given the variability within the autism spectrum, the inclusion of these data in future studies would allow for a more precise examination of sensory profiles and intra-group differences. Third, the decision to use a continuous measure rather than analyzing specific events may have impacted our intergroup comparisons. The observed fluctuations could have been influenced by internal variables, such as stress or fatigue, rather than the specific factors being studied. Finally, the lack of randomization in the presentation of stimuli also represents a limitation. As the audiovisual condition was systematically presented last, potential time-related effects such as habituation or fatigue cannot be fully ruled out. Future studies should implement a counterbalanced or randomized modality order to address this issue.

Although electrodermal reactivity provides an interesting marker for studying the modulation of autonomic functions in autism, it primarily reflects sympathetic nervous system activity and does not allow for a precise identification of the origin of this reactivity. Given the heterogeneity of sensory profiles in autistic individuals, future studies should complement physiological assessment with standardized behavioral measures (e.g., SP-2, SEQ) to examine potential associations between physiological and behavioral dimensions. The study of other aspects, such as emotions, in conjunction with sensory perception, seems relevant. Several studies have already addressed the issue of anxiety and identified an atypical electrodermal signature in autistic individuals (67, 68). These results support the hypothesis that the autonomic response may play an important role in the symptomatology of autism. Further investigations could be extended to all socio-emotional aspects, as well as to the processing of cognitive stimuli.

With further investigation, the use of this tool could be considered in clinical settings, provided that the findings are replicated and interpreted in combination with other assessment methods. For example, it could be used to monitor the effectiveness of therapeutic interventions (16, 17, 19). This indicator has the advantage of providing a quantitative and therefore objective measure. EDR could be used as a combined method alongside qualitative measures such as clinical interviews. While additional studies are needed to refine interpretation guidelines and determine the most effective clinical applications, the clear group differences observed in this study support the potential of EDR as a promising marker. Moreover, the conditions under which this research was conducted demonstrated the acceptability and feasibility of this method in clinical contexts.

## 5 Conclusion

The results of this study highlighted significant differences in autonomic regulation in autistic children, as evidenced by lower EDR at rest and during sensory stimulus processing compared to typically developing children. This study aligns with literature emphasizing the dysregulation of the ANS, increasingly recognized as a central element in understanding autism (67, 69, 70). Theoretically, the difference in ANS modulation could be considered within the framework of integrating it into existing theoretical models of sensory perception. Clinically, EDR thus emerges as an adequate tool for further research into the underlying mechanisms of sensory perception in autism.

## Data Availability

The dataset presented in this study can be found here: https://osf.io/e3fwv.

## Footnotes

1 Search conducted on December 23, 2025, on PubMed using the keywords “ autism OR PDD OR ASD AND sensory”.
