# Supplementary material for: Atypical modulation of electrodermal reactivity during exposure to graded unisensory and multisensory stimuli in autistic children and adolescents
Source: Front Psychiatry. 2026 Mar 19;17:1783075. doi: 10.3389/fpsyt.2026.1783075 (PMC13044047; doi:10.3389/fpsyt.2026.1783075)
Supplement: Supplementary file 1 [file DataSheet1.pdf]

Testing Procedure (estimate time – 1hour)

1. Completion of sociodemographic questionnaires (5-10 minutes)

Parents complete sociodemographic questionnaires before or during the testing session.

- For children recruited from schools, the questionnaires are completed online by parents before the experimental session.
- For autistic children, parents are invited to complete the questionnaires on site while the child undergoes testing.

2. Familiarization with the equipment (10-15 minutes)

A structured familiarization period is conducted prior to testing.
Children are introduced to:

- the testing room,
- the recording equipment (electrodes, connecting cables, display screen),
- the seating arrangement and task setup.

The procedure is explained step by step using simple, age-appropriate language. Children are allowed to touch the materials and ask questions.

For younger children, a model of the electrodes is demonstrated on a teddy bear or a plush panda before placement on the child's hand (Image 1).

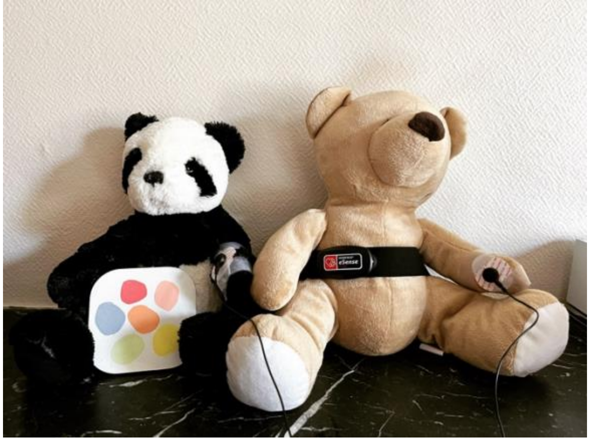

Image 1

Teddy bear and plush panda models

For autistic participants, an additional preparatory visit can be scheduled to reduce stress associated with novelty.

The testing environment is kept quiet and minimally stimulating, with limited visual and auditory distractions. Parents are allowed to remain in the room if this supports the child's comfort.

3. Administration of the Verbal IQ (15 minutes)

The session begins with administration of the Verbal Comprehension Index from the Wechsler Intelligence Scale for Children – Fifth Edition (WISC-V).

Standardized instructions are delivered according to the test manual.

4. Electrode placement (15 minutes)

Electrodes are placed on the palmar surface of the child's non-dominant hand. A plush model may be used for younger children. The investigator explains as follows:

> *"I am going to place these two small patches on your hand, here and here (the examiner points to the locations). It feels like a small bandage. When I put them on, you will need to keep your hand still. Please tell me if it feels too uncomfortable."*

The investigator then ensures that the child is comfortable before proceeding.

The investigator proposes establishing a simple gesture that the child can use to stop the task in case of discomfort:

> *"If at any point you would like to stop because it feels too uncomfortable, you can make this gesture (for example, raise your dominant hand)."*

5. Baseline recording (1 minute)

A baseline electrodermal recording is conducted prior to stimulus presentation (one minute).

Signal quality is checked in real time. If no anomalies are detected and the child appears comfortable, the experimental phase begins.

6. Sensory stimuli presentation (9 minutes)

Participants are exposed to nine sensory conditions (visual, auditory, and audiovisual stimuli at three intensity levels).
Each exposure lasts one minute and is presented consecutively, without breaks.

The standardized instructions given by the investigator are as follows:

<u>Visual condition:</u>

> *"Now, you are going to see videos showing the view from a train, first without sound. Each video lasts one minute. Try to look at the screen during the videos and avoid moving too much. There are three in total. You don't need to do anything, just watch."*

<u>Auditory condition:</u>

> *"Now, you will see a screen with a white cross in the middle. Try to focus on the cross while listening to the sounds through the headphones, without moving too much. Each audio recording lasts one minute, and there are three of them. You don't need to do anything, just listen."*

<u>Audiovisual condition:</u>

> *"Now, you will see videos of the view from a train with the sound at the same time. As before, try to look at the screen and avoid moving too much. There are three videos with sound, each lasting one minute. Again, you don't need to do anything, just watch and listen."*

If the child demonstrates understanding of the instructions, the task begins.

7. Monitoring of behavioral signs

During the test, the investigator continuously monitors signs of discomfort, agitation, or distress (*e.g.*, motor restlessness, attempts to remove the electrodes, verbal expressions of discomfort).

If significant discomfort is observed, the task must be paused or discontinued.

If the child does not maintain attention on the images or speaks while listening to the sounds, a reminder is given while physically pointing to the screen to help the child refocus on the task:

> *"You need to watch the images or listen to the sounds until the end."*

8. Conclusion of the session

At the end of the testing session, the electrodes are removed and children are invited to ask questions about the procedure.
